# COVID-19 et marqueurs immunologiques pertinents

**DOI:** 10.11604/pamj.2021.39.40.23481

**Published:** 2021-05-17

**Authors:** Brahim Admou

**Affiliations:** 1 Laboratoire d'Immunologie, Centre de Recherche Clinique, Centre Hospitalier Universitaire Mohamed-VI, Marrakech, Maroc,; 2 Laboratoire de Recherche Biosciences, Faculté de Médecine, Université Cadi Ayyad, Marrakech, Maroc

**Keywords:** COVID-19, formes sévères, marqueurs immunologiques, COVID-19, severe cases, immunological markers

## Abstract

Dans sa forme sévère, COVID-19 (corona virus disease-19) est caractérisée par diverses perturbations immunologiques, dominées par un largage massif de cytokines inflammatoires et de chimiokines, dont l'IL-6, le TNF-α, l'IL-1b, l'IFN-y et la protéine chimio-attractante monocytaire 1 (MCP-1), associés à une lymphopénie T CD3, T CD4 et T CD8. Ces anomalies sont significativement associées au syndrome respiratoire aigu sévère de COVID-19, et se normalisent en cas d'évolution favorable de la maladie. Combinés à d'autres paramètres biologiques (leucopénie, élévation de la CRP et des D-dimères, hyperferritinémie), les taux élevés d'IL-6 couplés à la lymphopénie T CD4 et CD8 représentent des critères de sévérité justifiant une admission en unité de soins intensifs, et sont également utiles pour le suivi des patients COVID-19.

## Abstract (en)

In its severe form, corona virus disease-19 (COVID-19) is characterized by various immunological abnormalities, dominated by massive pro-inflammatory cytokine and chemokine release, such as IL-6, TNF-α, IL-1b, IFN-y and monocyte chemoattractant protein-1 (MCP-1), associated with T CD3, T CD4 and T CD8 lymphopenia. These two abnormalities are significantly associated with COVID-19 acute severe respiratory syndrome. Conversely, these markers decrease during the favorable course of the disease. Coupled with other biological parameters such as leukopenia, increased level of CRP (C Reactive Protein), ferritin and D-dimers, high levels of IL-6 with CD4 and CD8 T cell lymphopenia may be considered as criteria of disease severity, justifying a rapid admission to the intensive care unit, and are also useful for patient monitoring.

## Commentary

Depuis son apparition, l'infection à SARS-COV-2, communément appelée COVID-19 (corona virus disease-19), a provoqué des groupes de maladies respiratoires graves similaires au syndrome respiratoire aigu sévère du coronavirus, nécessitant une admission en unité de soins intensifs (USI), avec une mortalité élevée [1]. Les niveaux de gravité de COVID-19 sont généralement classés en: 1. Forme légère (sans symptômes ni signes d'infection grave et critique; 2. Forme sévère: 2a. difficultés respiratoires, fréquence respiratoire ≥30cpm; 2b. SpO_2_ (saturation pulsée en oxygène) ≤93% au repos; 2c. PaO_2_/FiO_2_ (rapport pression partielle artérielle et fraction inspirée en oxygène) ≤300mmHg]; 3. Forme critique: 3a. insuffisance respiratoire; 3b. choc; 3c. défaillance multi-viscérale [2].

Le rôle du laboratoire de biologie médical dans le diagnostic différentiel des formes graves de COVID-19 n'a pas été définitivement établi [3]. En effet, le laboratoire de microbiologie confirme d'abord le diagnostic par PCR, et ceux de biochimie et d'hématologie assurent les paramètres biologiques fondamentaux afin d´évaluer les critères de gravité de la maladie. Le laboratoire d'immunologie est également appelé à jouer un rôle important, notamment pour aider à classer les cas légers, graves et critiques de COVID-19, et par conséquent, accompagner le clinicien tout au long du processus de prise en charge de la maladie.

**COVID-19 et orage cytokinique:** l'infection par le SARS-CoV-2 se caractérise par une surproduction de cytokines pro-inflammatoires et de chimiokines, principalement l'interleukine 6 (IL-6) et le facteur de nécrose tumorale α (TNF-α), l'IL-1b, l'interféron (IFN)-y et la protéine chimio-attractante monocytaire 1 (MCP-1) [4]. D'autres anomalies biologiques peuvent prédire la sévérité ou l'évolution potentielle vers une maladie grave, comme une activation anormale de la coagulation, la leucopénie, des niveaux élevés de CRP (protéine C réactive), de la ferritine, des D-dimères, des aminotransférases, dU lactate déshydrogénase (LDH) et de la créatine kinase [3].

Des études antérieures ont montré que le niveau élevé de cytokines inflammatoires s´associe à une infection pulmonaire sévère illustrée par la détresse respiratoire, la défaillance multi-viscérale et les conséquences néfastes de l'infection par le SARS-CoV-2 [4,5], ce qui suggère que l'ampleur de la tempête cytokinique est associée à la gravité de la maladie. En outre, chez les personnes infectées par SARS-CoV-2, l´IL-6, et le TNF-α augmentent pendant la maladie et diminuent pendant la convalescence [6]. De plus, il a été démontré que l'augmentation élevée du taux d'IL-6 et des D-Dimers est étroitement liée à la survenue des formes graves de COVID-19 chez les adultes, et que leur détection combinée présente une plus grande spécificité et sensibilité pour prédire de façon précoce les formes sévères, ce qui leur confère un intérêt clinique important [3].

**COVID-19 et anomalies des lymphocytes T:** la dérégulation de la réponse immunitaire, en particulier des lymphocytes T, semble être fortement impliquée dans le processus pathologique lié à COVID-19 [7]. En fait, le SARS-CoV-2 semble infecter directement les lymphocytes, entraînant leur destruction ou leur dysfonctionnement avec un déclin aigu [3,8]. D´ailleurs, les lymphocytes expriment le récepteur ACE2, ce qui les rend une cible directe du virus, donc leur altération [9] et représenterait un facteur important d'exacerbation des symptômes de l´infection. La lymphopénie peut ainsi être considérée comme un indicateur efficace et fiable de la gravité et donc de l'admission des patients en USI [8]. Il a été démontré que le syndrome respiratoire sévère inaugurant la maladie s´associe à une lymphopénie T CD4 et CD8, et les patients nécessitant une admission en USI présentent des niveaux significativement plus élevés d'IL-6 et de TNF-α et significativement plus faibles de lymphocytes T CD4 et CD8 [6,10].

Le rôle des lymphocytes T dans la modulation des réponses immunitaires au cours d´une infection virale est important, et une perte de ceux-ci pendant l´infection par COVID-19 peut entraîner une aggravation des réponses inflammatoires, tandis que leur restauration peut les améliorer [5]. En accord avec cette hypothèse, il a été montré que 4 à 6 jours après le début de la maladie, et au moment où le nombre de lymphocytes T chute à son niveau le plus bas, les niveaux de cytokines atteignent leur maximum. À l'inverse, la restauration de leur nombre est associée à une diminution des taux sériques d'IL-6, de TNF-α et d´IFN-γ [5].

**COVID-19 et exploration immunologique:** sur la base des paramètres immunologiques les plus pertinents mentionnés ci-dessus (cytokines inflammatoires, sous-populations de lymphocytes T), la contribution du laboratoire d'immunologie est importante pour évaluer la gravité des patients COVID-19 [2,7]. Le dosage des cytokines, en particulier de l'IL-6, est aujourd'hui disponible même séparément sur les automates d´immunoanalyse, tandis que les autres profils cytokiniques peuvent être explorés soit par une méthode ELISA, soit à l´aide de biotechnologies sophistiquées comme le système luminex et la cytométrie en flux, permettant une mesure quantitative d´un panel cytokinique plus large. L'évaluation des sous-populations lymphocytaires T CD3+, CD4+ et CD8+ nécessite une procédure simple de phénotypage, réalisable sur des mini-cytométres, disponibles même dans les laboratoires à ressources limitées, grâce notamment aux programmes de gestion de l´infection à VIH.

Des plateformes de cytométrie en flux plus sophistiquées permettent un immunophénotypage complet des sous populations, en explorant également les cellules T naïves et mémoires ainsi que les cellules T régulatrices. En effet, les premières données sur les perturbations des sous-populations lymphocytaires chez les patients COVID-19 graves rapportent une augmentation des lymphocytes T auxiliaires naïfs et une diminution des lymphocytes T mémoires et régulateurs [7]. En utilisant une courbe ROC pour comparer les cas critiques et les cas de COVID-19 guéris, Xu *et al*. ont montré que 559, 235 et 104 cellules T CD3, CD4 et CD8 respectivement représentaient des valeurs d'alerte, en dessous desquelles il y avait un risque significativement plus élevé de décès [2]. Cependant, toute lymphopénie T peut être accentuée par d'éventuels déficits immunitaires primitifs ou acquis, potentiellement révélés par COVID-19, et doit donc être prise en compte en premier lieu lors de l'interprétation des valeurs du phénotypage.

Enfin, malgré la contribution pertinente des biomarqueurs immunologiques présentés dans cette brève revue, le domaine des investigations immunologiques est prometteur et devrait apporter des réponses à d'autres questions d´actualité en rapport avec COVID-19, notamment les différents profils de cytokines, de chimiokines, l'importance des lymphocytes T régulateurs dans la modulation et le contrôle de la réponse immunitaire, la cinétique de la réponse humorale, y compris l´activité neutralisante des anticorps anti-SARS-CoV-2.

## Conclusion

Au cours de l'infection à SARS-CoV-2, outre les paramètres cliniques et biologiques classiques, le dosage des cytokines inflammatoires, principalement l'IL-6, ainsi que l´étude des sous populations lymphocytaires T CD3, CD4 et CD8 doivent être systématiquement planifiés pendant la prise en charge des formes sévères de COVID-19. L´étude de ces marqueurs permet de définir les formes graves nécessitant une admission rapide en unité de soins intensifs, et sont également utiles pour le suivi des patients. Ces investigations nécessitent une démarche qualité et une collaboration étroite entre immunologistes et cliniciens en faveur des efforts immenses déployés contre cette pandémie.
